# Allele specific CAPS marker development and characterization of chalcone synthase gene in Indian mulberry (*Morus* spp., family Moraceae)

**DOI:** 10.1371/journal.pone.0179189

**Published:** 2017-06-22

**Authors:** Vivek Arora, M. K. Ghosh, Soumili Pal, Gaurab Gangopadhyay

**Affiliations:** 1 Division of Plant Biology, Bose Institute (Main Campus), Kolkata, India; 2 Central Sericultural Research & Training Institute, Berhampore, India; National Institute of Plant Genome Research, INDIA

## Abstract

Chalcone synthase (CHS) is an essential enzyme in the phenylpropanoid pathway that catalyzes the first step in flavonoid biosynthesis in plants under diverse environmental stress. We have used CHS as a candidate gene in mulberry and developed Single Nucleotide Polymorphism (SNP) based co-dominant Cleaved Amplified Polymorphic Sequence (CAPS) marker associated with the *CHS* locus. The segregation pattern of the marker was studied in an F_1_ population derived from a hybridization program between two mulberry genotypes showing polymorphism for the *CHS* locus. Differential CHS activity of the recombinants has been correlated with the segregation pattern of the marker. Homology modelling and docking studies are performed for both the identified *CHS* alleles and correlated with respective CHS activity. Phenotyping of Powdery Mildew infected F_1_ population indicated a probable association with the CAPS marker.

## Introduction

Mulberry (*Morus* spp., family Moraceae) foliage is the only forage for the silkworms (*Bombyx mori* L.). Hence it is the most important plant from sericulture point of view. Mulberry essentially is a fast-growing perennial tree, maintained as short or medium bushes by repeated pruning and conventional propagation is by vegetative means through stem cuttings [1]. In recent years the cultivation of mulberry is declining very fast due to preferences for growing of cereals and other high-value crops in the shrinking arable land. Additionally, several environmental stresses including diseases caused by fungi, bacteria and viruses are limiting its yield. Stress-related yield loss of mulberry ranges from 50% to 60% [2–3]. The solution to this problem lies in the development of superior mulberry genotypes having tolerance to biotic and abiotic stresses vis-à-vis higher yield. Like most of the tree crops, mulberry is extremely heterogeneous and out breeding in nature. The dioecious nature coupled with long juvenile period and high heterozygosity acts as the significant impediment to developing inbred lines in mulberry [4].

The challenges in the mulberry breeding lie in the identification of suitable markers, be it morphological or molecular, and to link those with significant traits so that they are helpful for early screening of promising recombinants in Marker Assisted Selection (MAS) program [1]. Allele-specific candidate gene-based molecular markers possess distinct advantages over other markers in any breeding programme because they detect Single Nucleotide Polymorphisms (SNP). However, development of this type of marker necessarily requires prioritisation of candidate genes [5].

In the present study, we are proposing chalcone synthase as an important candidate gene for the mulberry breeding programme. Chalcone synthase is a well-studied type III polyketide synthase and is one of the essential enzymes which channel the flux of phenylpropanoid pathway towards the biosynthesis of flavonoids [6]. The phenylpropanoid pathway in plants plays a crucial role in the production of many physiologically active secondary metabolites like flavonoids, lignin, isoflavonoids and anthocyanins [7]. Flavonoids, in particular, play a significant role in various biological processes like pollination, floral pigmentation and nitrogen fixation. Additionally, they are produced in response to stress, UV-radiation, pathogens and insects [8–13]. CHS catalyzes the condensation of three molecules of malonyl-coA and one molecule of p-coumaroyl-CoA, yielding naringenin chalcone [14], which is the precursor of all flavonoids. In a recent study, nine putative genes behind anthocyanin biosynthesis including CHS have been identified in mulberry [15].

The present study aims to assess *CHS* as a probable candidate gene in mulberry and mining it for Single Nucleotide Polymorphisms (SNP) to develop suitable SNP-based molecular markers. We have validated the developed marker in an F_1_ recombinant population derived from a hybridization programme conducted by Central Sericulture Research and Training Institute (CSR&TI) at Berhampore, India. The hybridization was done between a high yielding genotype (widely grown by the mulberry growers in several parts of India) and an open pollinated selection of a landrace (with disease resistance) to develop superior mulberry genotype. Homology modelling and stereo chemical analysis of mulberry CHS and molecular docking study of *CHS* alleles will help to foster future marker assisted breeding of mulberry with an aim to combat biotic or abiotic stress. The phenotyping of Powdery Mildew infected F_1_ population was also done to evaluate the probable association of the CAPS marker with degree of disease severity.

## Materials and methods

### Plant materials

The primary study materials were three genotypes of mulberry, viz. V1 (MI-0008), Gen1 and Kajli-OP (OP—Open Pollinated) [16]. The genotype Gen1, awaiting registration as a new mulberry variety, is a clonal selection from the F_1_ population of the hybridization between V1 (♂) and Kajli-OP (♀). Besides, fifty-five random recombinants were selected from the same F1 population (original sample size one hundred and eighty-four). Additionally, ten mulberry varieties (S30—MI-0046, Kajli—MI– 0068, C763—MI– 0124, C2038 –no accession number, C776—MI-0158, C2028—no accession number, S1635—MI– 0173, Bombai Local—MI– 0112, S1—ME-0065, CF_1_10—no accession number) were also used for validation of the CAPS marker. All the recombinants and genotypes used are maintained in the field of Central Sericulture Research and Training Institute (CSR&TI) at Berhampore, WB, India.

### Identification of chalcone synthase gene and designing of primers

The nucleotide sequence of *Morus notablis* chalcone synthase (*CHS*) gene (GenBank: KT630885) was downloaded from *Morus* Genome Database and was used for multiple sequence alignment with the *CHS* genes reported from other trees and dicotyledonous plants. The conserved sequences were identified for designing primers to amplify partial genomic sequence (MCHS_CAPS);partial coding sequence (MCHS1)and full-length coding sequence (MCHS2) of CHS from the three varieties (Table 1).

**Table 1:** List of primers.

Primer name	Primer sequence	T_a_ (°C)	Amplicon size (bp)
MCHS_CAPS (F)	5´-CTATGGCGCCGAATAACGTG	60	921
MCHS_CAPS (R)	5´-CCAGCGAAACAACCTTGGTG		
MCHS1 (F)	5’-ATGTGTAGCTATGATGCTCC	60	254
MCHS1 (R)	5’-CCAGCGAAACAACCTTGGTG		
MCHS2 (F)	5’-ATGGCGCCGAATAACGTGTC	60	1200
MCHS2 (R)	5’-CTATGCAACAATGGGGACGC		

### RNA extraction and preparation of cDNA

Total RNA was extracted from the primary study materials- the two parents and the selected hybrid from the leaves (100 mg fresh wt) of the three mulberry genotypes using SpectrumTM Plant Total RNA kit (SIGMA). RNA was quantified spectrophotometrically using a Nanodrop Spectrophotometer (Thermo Scientific) and resolved on 0.8% (w/v) agarose gel to ascertain the RNA quality. First strand cDNA was synthesized using 1μg of total RNA using QuantiTect® Reverse Transcription kit following the manufacturer’s (QIAGEN) protocol; used as the template for subsequent PCR amplification reactions.

### DNA extraction

Genomic DNA was isolated from newly emerging sprouts of each genotype using Qiaquick DNeasy plant mini kit (Qiagen) following the manufacturer’s protocol. Subsequently, DNA was quantified using Nanodrop Spectrophotometer (Thermo Scientific) and resolved in 1.0% (w/v) agarose gel. DNA was isolated from pooled samples of five clonal plants of each genotype for the two parents (V1 and Kajli-OP) and the selected hybrid (Gen 1). Furthermore, DNA was isolated from each F_1_ recombinant plant (fifty-five) with three biological replicas. Additionally, DNA was isolated from ten high yielding genotypes for the validation experiment.

### PCR amplification and cloning of amplicons

For polymerase chain reaction the cDNA and genomic DNA were used as templates using respective pairs of primers (Table 1). The reaction volume of PCR reactions was 25 μl. It contained 2.5 μl of 10X NH4 buffer, 1.25 μl of 50mM of MgCl_2_, 2.5 μl of 200 μM dNTP, 0.5 U of Taq polymerase (BIOLINE), 1.0 μl 100 μM primer, 2 μl of DNA template (final concentration 25 ng) and PCR grade water for making up the volume. The reactions have been performed using MJ Research Thermal Cycler. The PCR programming was: initial denaturation of 2 min at 94°C followed by 29 cycles of 1 min denaturation at 94°C, 1 min at annealing temperature (60°C) and 1 min extension at 72°C followed by 20 min final extension at 72°C. The amplified products were resolved on 1.6% (w/v) agarose gels (1X TAE, 7 V/cm). The amplicons of the three genotypes were cloned using TOPO TA cloning Kit (Invitrogen) and the replica clones were sequenced at the DNA Sequencing Facility, South Campus, Delhi University (New Delhi) and CIF, Bose Institute, Kolkata, India.

### Multiple sequence alignment and development of CAPS marker

The sequences of the amplicons obtained from the three genotypes were used as queries for BLAST searches in NCBI and *Morus* Genome Database (http://morus.swu.edu.cn/morusdb/). The sequences obtained from the BLAST hits were subjected to multiple sequence alignments using Clustal Omega to ascertain the presence of SNP. SNP identified from partial genomic sequence was successfully converted to CAPS (Cleaved Amplified Polymorphic Sequence) marker using SNP2CAPS tool. The first input file was a FASTA formatted file that contained the multiple alignments of the particular nucleotide sequence from the three genotypes. The second input file contained the data of different restriction enzymes downloaded from restriction enzyme database REBASE (http://rebase.neb.com/). The CAPS marker and the sequence of the locus were submitted to NCBI database (GenBank: KM210515). We tested the developed marker in the F_1_ recombinant population and the parents. For this, the partial genomic sequence of *CHS* locus was amplified using the specific primer pair (F: 5´-CTATGGCGCCGAATAACGTG, R: 5´- CCAGCGAAACAACCTTGGTG) and digested with EcoRI restriction enzyme after PCR product purification.

### Amino acid sequence analysis

The full-length coding sequence of mulberry *CHS* gene was analysed and converted to amino acid sequence using Expasy translate tools (http://www.expasy.org/). The protein sequence of mulberry CHS obtained was used as query for BLASTP search to identify closely related CHS homologs.

### Chalcone synthase assay

The activity of chalcone synthase was assayed spectrophotometrically [17] where the three replicate samples each of fifty-eight individuals were used for enzyme assay. The enzymes were extracted at 4°C by homogenising frozen harvested leaves (0.4g) in 1 ml of 0.1M borate buffer (pH 8.8) containing 1mM 2-Mercaptoethanol with a homogenizer (Polytron). The homogenates were treated with 0.1g of Dowex l × 4 for 10 min. The cell debris and the resins were removed by centrifugation at 15,000 rpm for 10 min. Retreatment of the supernatant was done with 0.2g of Dowex l × 4 for 20min. The resin was removed by centrifugation at 15,000 rpm for 15 min. The resultant supernatant was used for chalcone synthase assay. The assay was performed with 100 μl of enzyme extract mixed with 1.89 ml of 50 mM Tris-HCL buffer, pH 7.6, containing 10mM KCN. The enzyme reaction was allowed to proceed for 1 min at 30°C after adding 10mg of chalcone to 10 μl of ethylene glycol monomethyl ether. Chalcone (4, 2’, 4’, 6’–tetrahydroxy chalcone) was prepared from naringenin [18]. Pure Naringenin was procured from Sigma-Aldrich (Germany). The enzyme activity was determined by measuring absorbance at 370 nm and expressed in katals.

### Homology modelling and structural analysis

The protein model of mulberry CHS was generated using the SWISS-MODEL [19] package provided by the Swiss-PDB viewer program based on the crystal structure of CHS (PDB ID: 4WUM) as the template. The model quality was assessed using PROCHECK [20]. The stereochemical stability of the model was checked, and Ramachandran plot for the model was obtained.

### Molecular docking

The homology model created for mulberry CHS was uploaded onto SWISS-Dock. The mol2 file of malonyl-CoA was uploaded onto the SWISS-Dock in the ligand selection tab. The mol2 file for malonyl-CoA was generated using UCSF Chimera. The.sdf file for malonyl-CoA was obtained from PDB (PDB code: MLC). This.sdf file was then opened with UCSF Chimera, and the hydrogen atoms were added using Tools/Structure editing/ AddH menu and the file was saved in the mol2 format [21]. All protein structures are prepared with PYMMOL (DeLano Scientific, http://www.pymol.org).

### Statistical analysis

Student’s t-test was performed to analyze the level of significance between the mean enzyme activities of homozygous and heterozygous plants for the *CHS* locus.

### Phenotyping of Powdery Mildew infected F_1_ mulberry population

**Fig 1:**
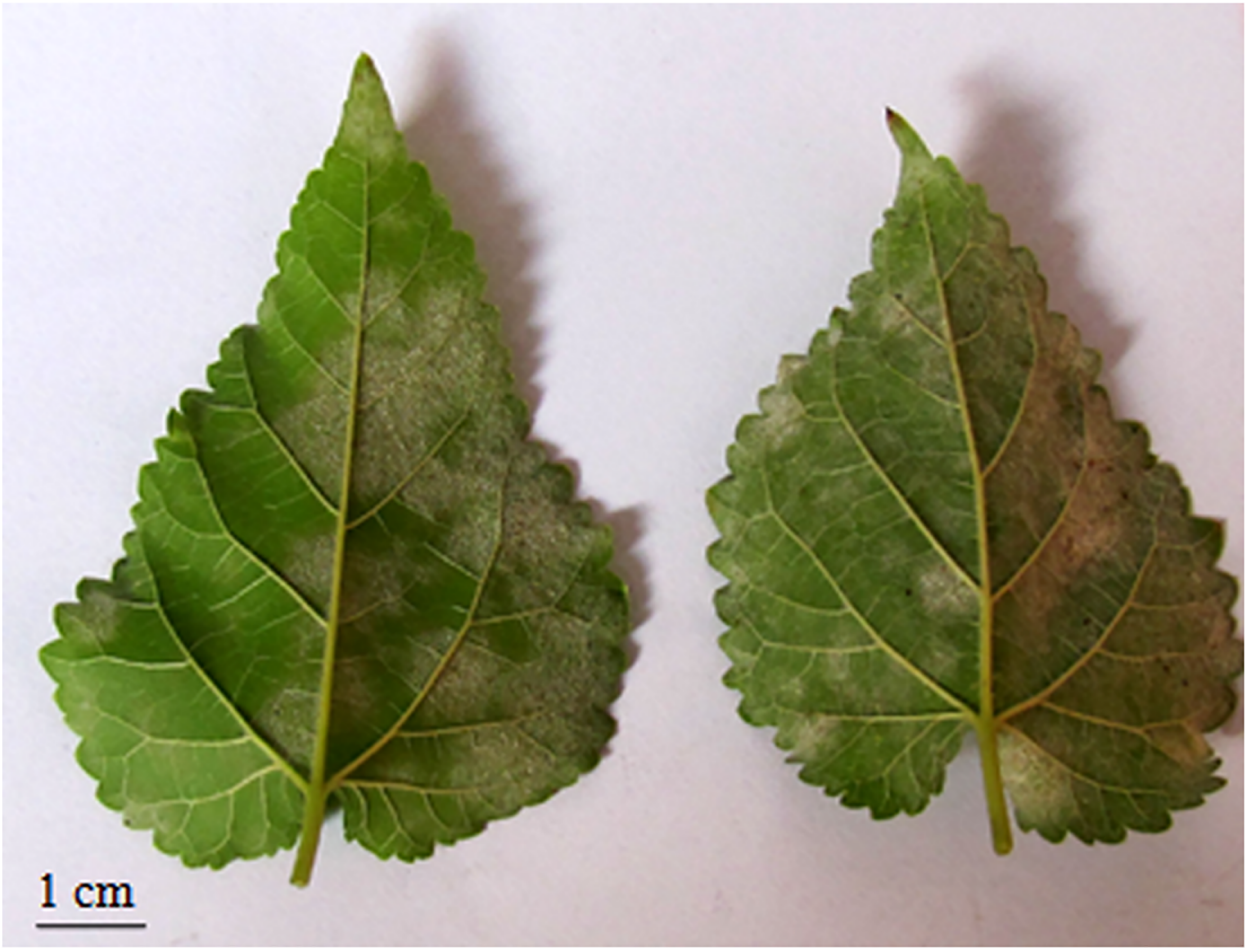
Powdery Mildew- infected mulberry leaves.

F_1_ recombinant mulberry population including theparents were screened for infection with powdery mildew and assigned to different degree of disease severity.The individual plants of the population (one month after the cuttings were established in pot culture) were inoculated by gently rubbing detached powdery mildew infected leaves onto the leaves of the healthy plants. Plants were allowed to grow at a temperature range of 22±2°C and 75–85% humidity. The plants developed powdery mildew symptoms on inoculated leaves after 10–15 days. The morphological characteristics of the pathogen and symptoms on the leaves of inoculated plants were similar to that of the naturally infected plants. The abaxial surfaces of leaves were infected with white mycelia mat (Fig 1). Corresponding chlorotic spots were observed on the adaxial leaf surfaces. The degree of disease severity ranged from countable whitish spots to mycelial mat covering most of the abaxial surface (Table 2).After isolating the sporulating mycelia,the fungal structures were microscopically examined.

**Table 2:** Disease scoring of Powdery Mildew infection.

Disease score	Symptom on abaxial surface	% of leaf area covered
0	No disease on leaf	0%
1	Small white spots	<10%
2	Circular white spots	10–20%
3	Circular white spots	21–40%
4	Circular white spots	41–60%
5	Circular to irregular white patches	>60%

## Results

### Identification and characterization of *CHS* gene

**Fig 2:**
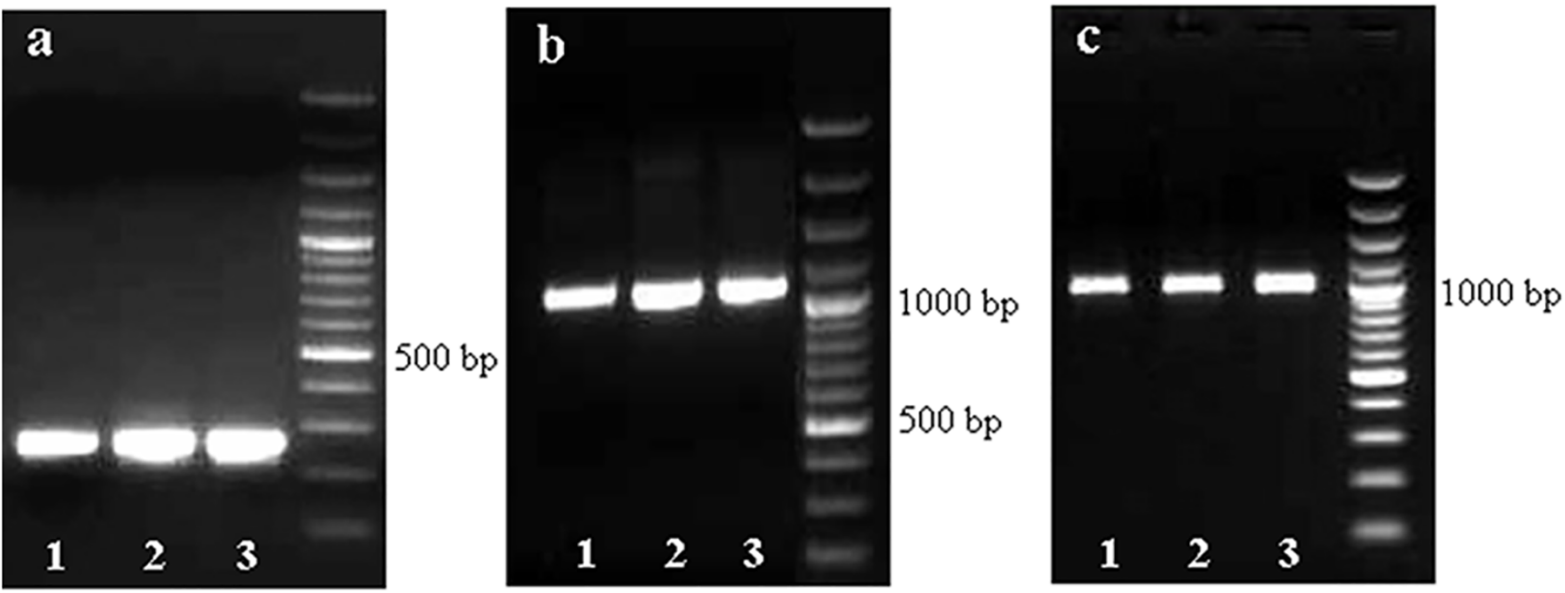
Target amplicons of partial CDS (a), full length CDS (b) and partial genomic sequence (c) of *CHS* in mulberry.

**Fig 3:**
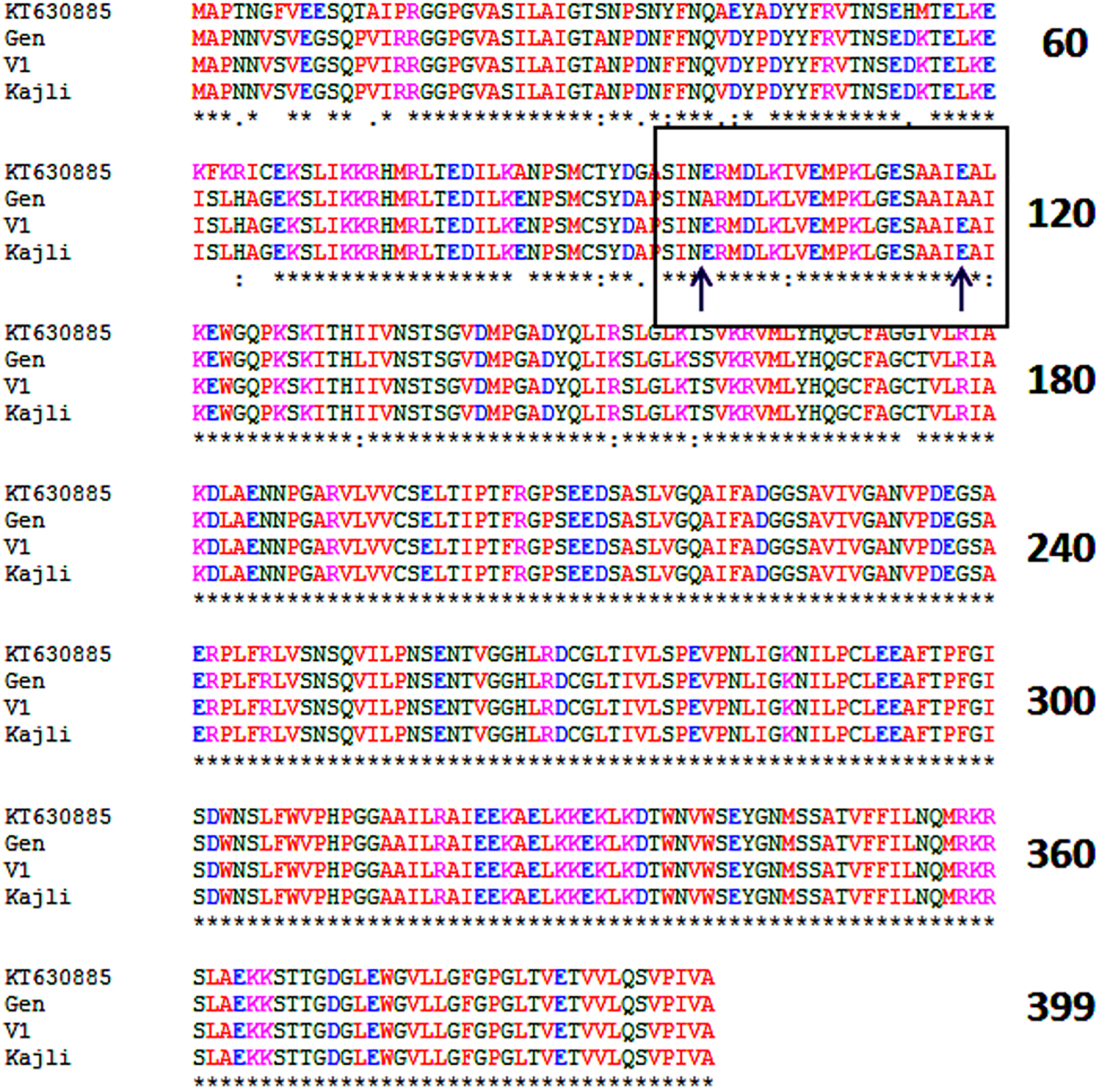
Multiple sequence alignment of CHS protein in three mulberry genotypes under study along with the Chinese mulberry *CHS* (GenBank: KT630885). The amino acid substitutions at 99^th^ and 118^th^ position has been marked with a rectangle and pointed by arrows.

Target amplicons of partial and full-length coding sequence of *CHS* gene were obtained in the three varieties. The size of the amplicons was 254 bp and 1200 bp respectively (Fig 2A and 2B). Similarly, desired amplicon of 921 bp was obtained from the partial genomic sequence (Fig 2C). The full-length sequence of *CHS* gene was converted to the amino acid sequence in three varieties. The sequences were also used for conserved domain search [22–25], and it showed that the *CHS* gene showed highly conserved amino acids in the active site as well as in the substrate binding site in the three varieties. However, the sequence of the recombinant variety (Gen1) showed two particular SNPs in the malonyl coA binding site leading to a significant amino acid change from glutamic acid to alanine in the 99th and 118th position (Fig 3).

### CAPS marker development

**Fig 4:**
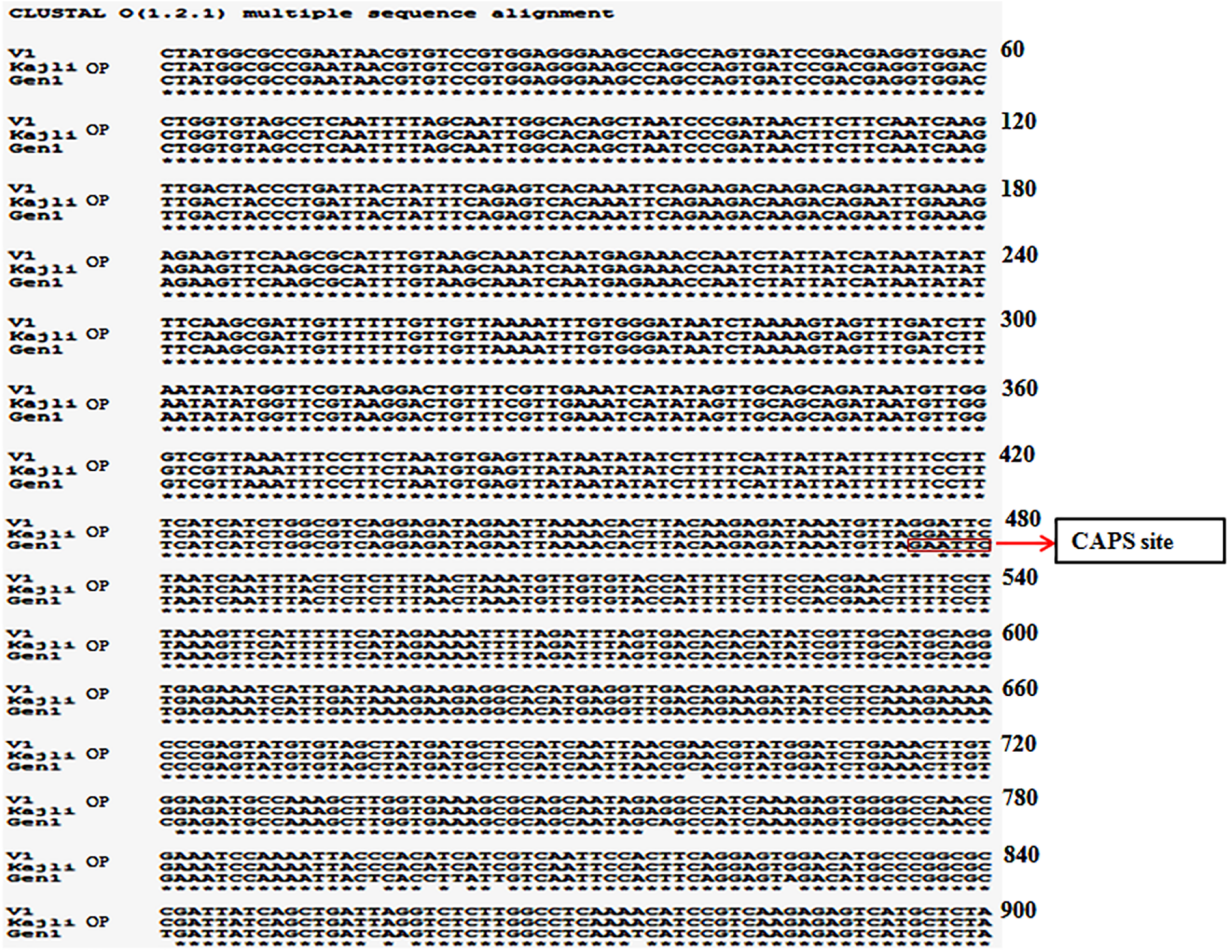
Multiple sequence alignment of the partial chalcone synthase genomic sequence of three mulberry genotypes. SNP with restriction enzyme (EcoR1) site is marked with rectangle.

The partial genomic sequences of *CHS* locus revealed the presence of several SNPs in the nucleotide sequences of the three varieties. Furthermore, the presence of EcoRI recognition site (GAATTC) was noticed in the sequence of Gen1, the recombinant variety (Fig 4).

### Screening of the CAPS marker in the F_1_ population

**Fig 5:**
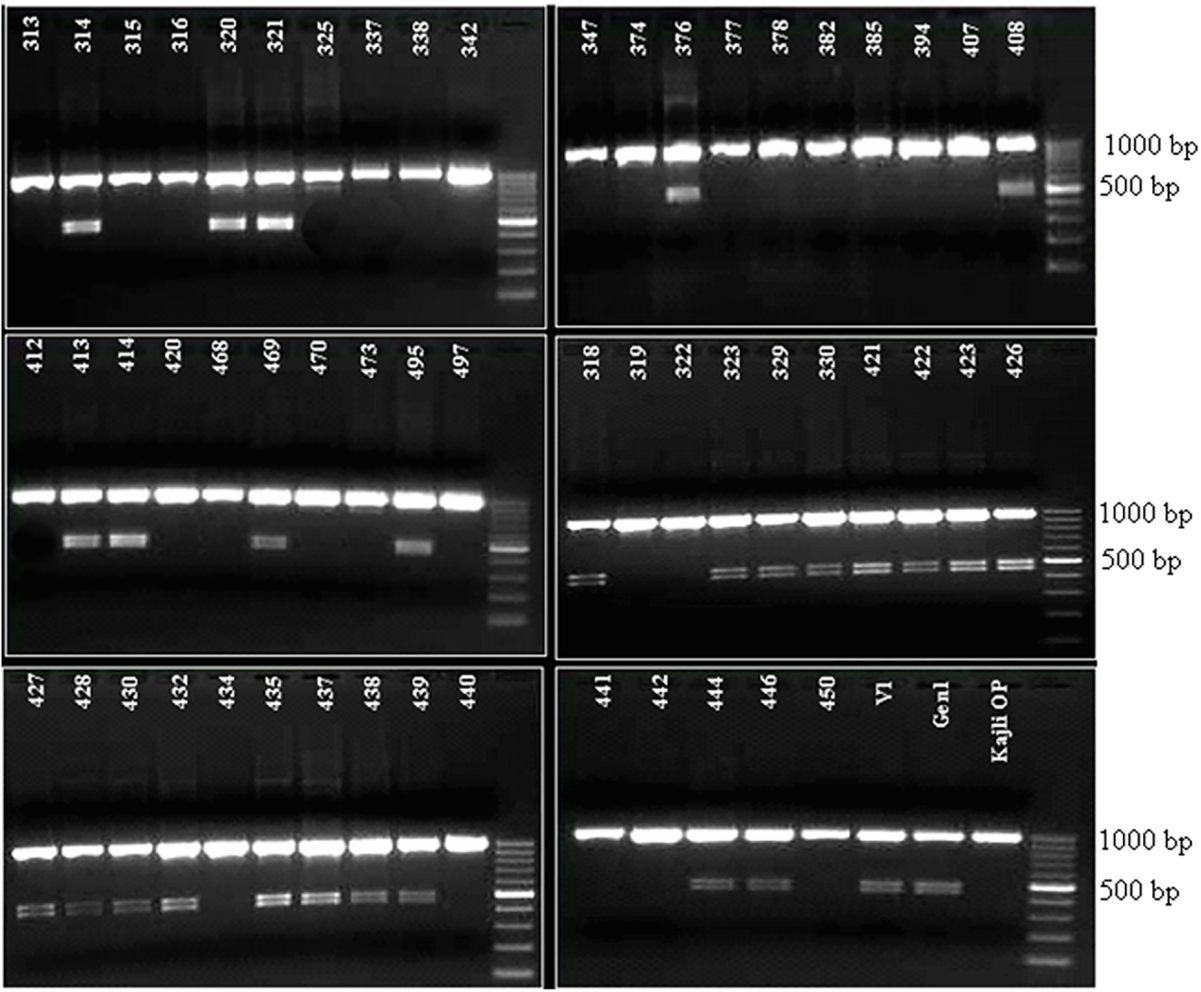
Segregation analysis of EcoR1 derived CAPS marker of *CHS* allele in random F_1_ recombinant population (55) and the male (V1), female (Kajli-OP) and the selected hybrid (Gen1). Digits in the figure represent the plant identifier number.

The restriction digestion profile of the twenty-eight out of fifty-five recombinants showed only one uncut DNA fragment of ~1 kb while the other twenty-seven recombinants showed three restriction-digested DNA fragments one at ~1 kb and other two at ~500 bp. Thus the CAPS marker differentiated the population into two distinct groups (Fig 5), the first and second being the homozygotes and heterozygotes, respectively, for the *CHS* locus. The marker also indicates that the male parent (V1) and the selected hybrid (Gen1) are heterozygous for the *CHS* locus while the female parent (Kajli-OP) is homozygous (Fig 5).

### Screening of the CAPS marker in differentmulberry varieties

**Fig 6:**
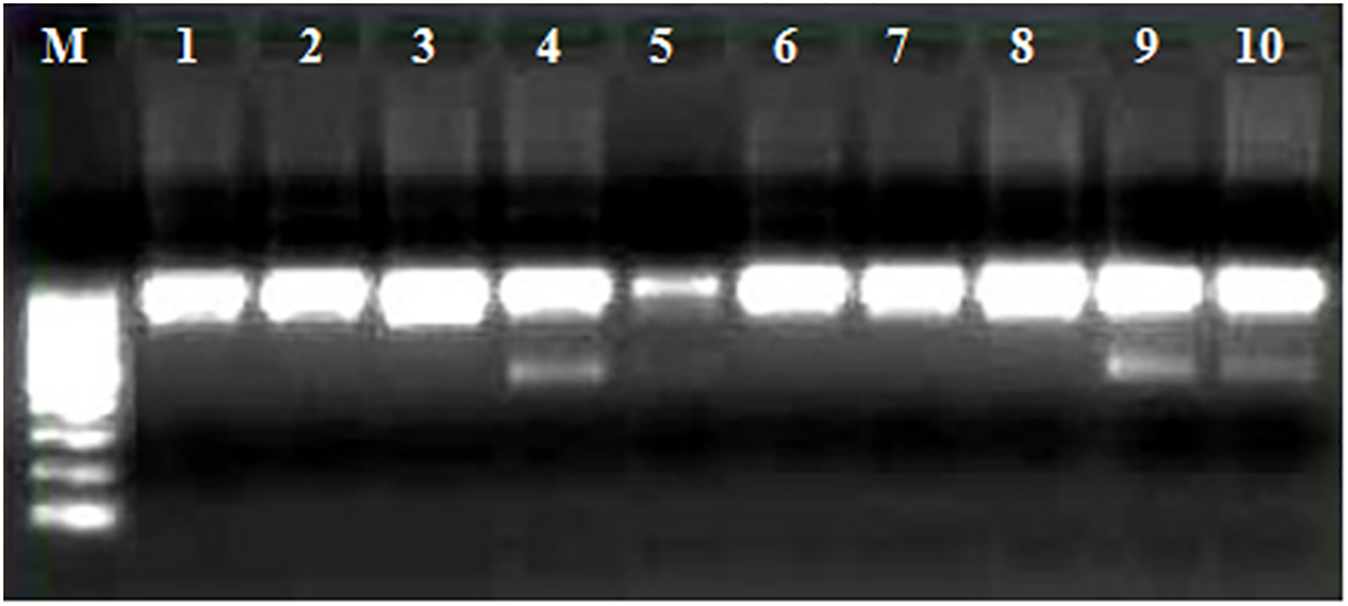
Screening of diverse mulberry varieties using the CAPS marker. M–DNA size marker, 1 –C776, 2—S30, 3 –C763, 4 –CF_1_10, 5 –C 2038, 6 –S1635, 7 –S1, 8 –C2028, 9 –Bombai Local, 10 –Kajli.

The restriction digestion profile showed that the additional DNA fragments indicating heterozygosity were noticed in four varieties (CF_1_10, C 2038, Bombai Local and Kajli), while the rest were homozygous for *CHS* locus (Fig 6).

### Chalcone synthase enzyme (CHS) activity

**Fig 7:**
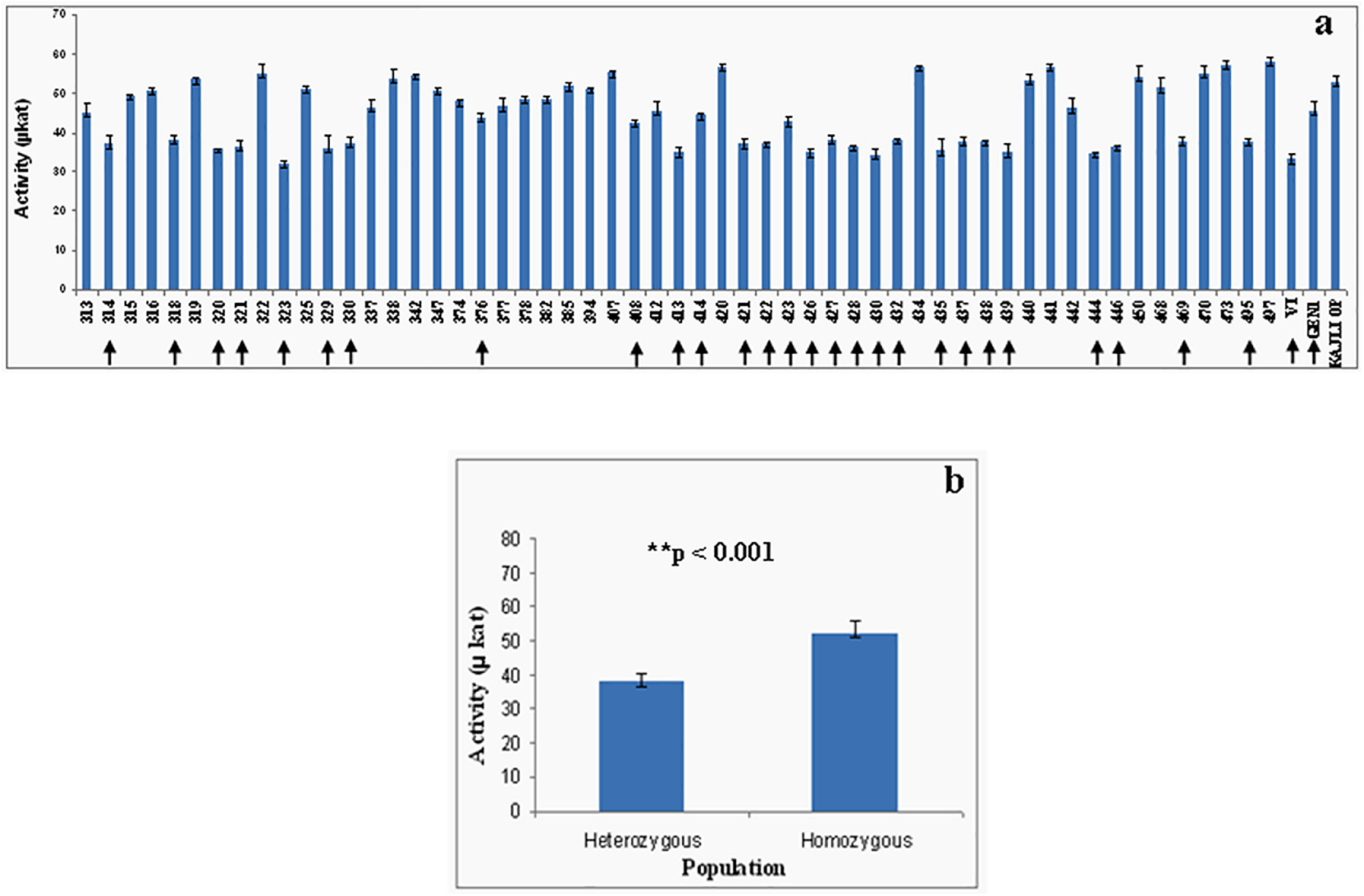
Assay of chalcone synthase enzyme. (a) CHS activity of the F_1_ recombinants along with the male (V1), female (Kajli-OP) and the selected hybrid (Gen1). The heterozygous recombinants for the *CHS* locus identified by the CAPS marker are arrow marked. (b) Mean CHS enzyme activity of the homozygous and heterozygous plants and their statistical analysis (Student’s t-test).

The CHS enzyme activity of two parental genotypes, the selected recombinant and the fifty-five recombinants was found to vary within a range from 33.6μkat– 57.6μkat.The CHS activity results of the recombinants were further found to lie in two broad groups–with one group having enzyme activity in the range of 47.1μkat- 57.6μkat with mean CHS activity of 52.46μkat, and the other group having CHS activity in the range 33.6μkat– 43.8μkat and mean enzyme activity of 37.74μkat. The CHS activity showed a significant difference between the mean enzyme activities of homozygous and heterozygous plants for the CHS locus (Fig 7).

### Homology modeling and stereo chemical analysis of mulberry CHS

**Fig 8:**
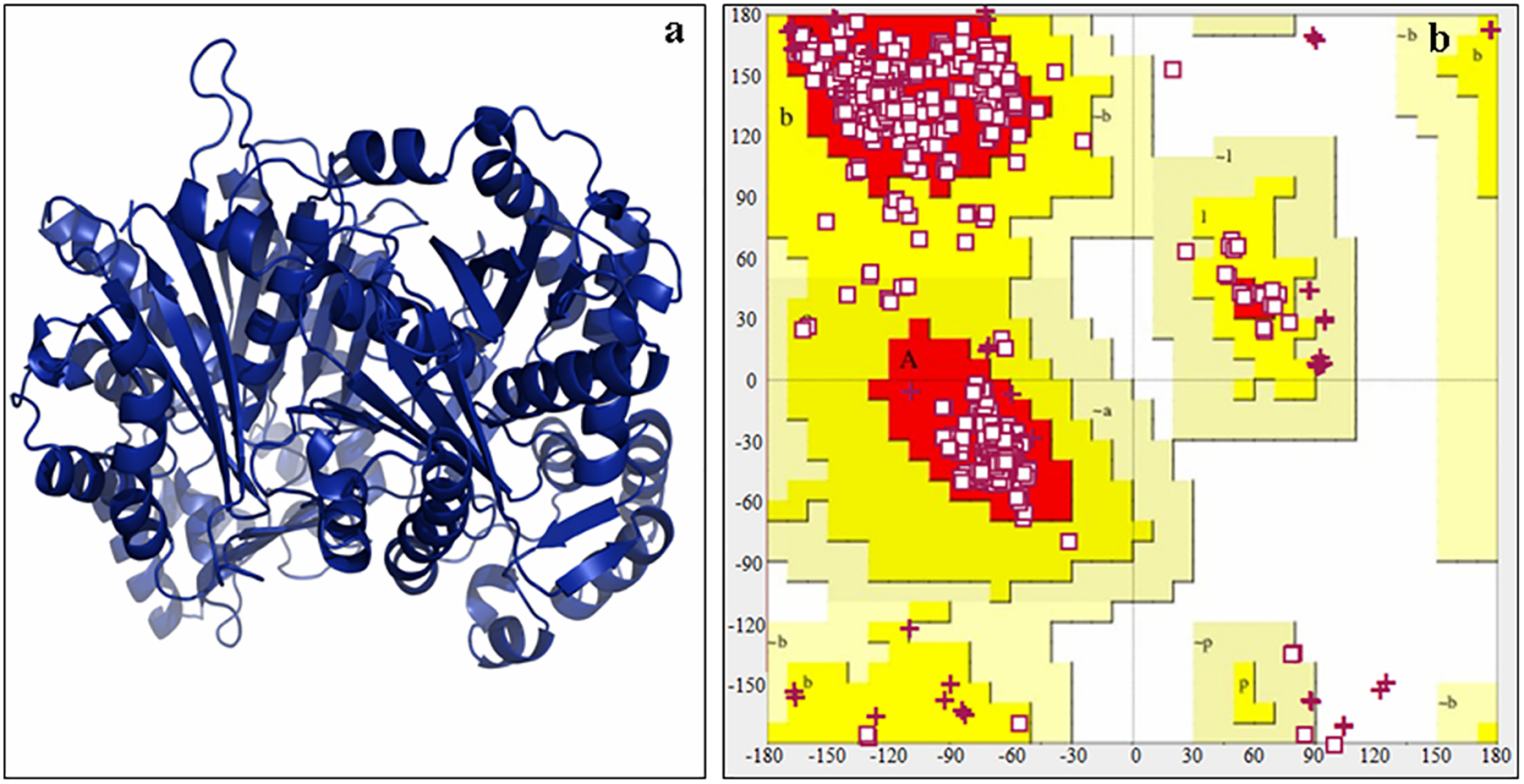
Protein model and stereochemical study of mulberry CHS. Predicted model (a) and Ramachandran plot (b) of mulberry CHS from recombinant variety, Gen1.

Homology modelling of both the *CHS* alleles was carried out using CHS PDB ID: 4WUM as a template. The protein model was developed using SWISS-MODEL program. The stereo chemical analysis of the mulberry CHS protein model (Fig 8A) was performed using the PROCHECK server (http://services.mbi.ucla.edu/SAVES/). The Ramachandran plot analysis of the generated protein model showed that 95.6% of the amino acid residues lied in the most favourable region while 3.1% residues lied in the allowed region and the remaining 1.3% residues fall in the outlier region. The result of PROCHECK analysis showed that no residue has phi/psi angles in the disallowed region suggesting the acceptability of the Ramachandran plot for mulberry CHS protein (Fig 8B).

### Molecular docking study of CHS alleles

**Fig 9:**
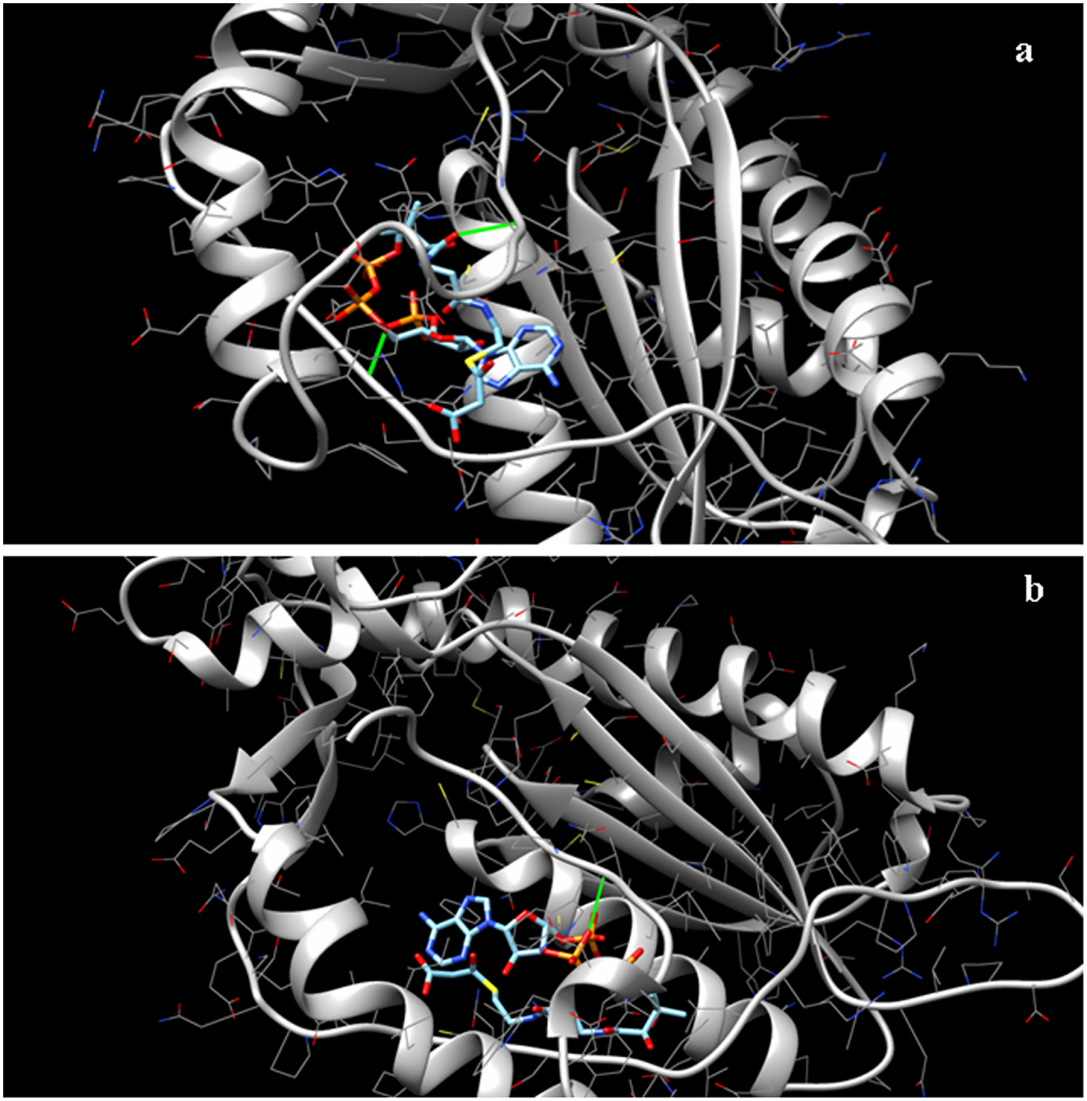
Docking prediction of ligand (malonyl coA) with templates (protein models of *CHS* alleles). Mulberry variety V1 (a), Gen 1(b); green colour indicates differential interaction of the ligand with templates.

The docking study was done to validate the difference in the enzyme activity of the two *CHS* alleles. Two amino acid substitutions as mentioned earlier (Fig 3) in the malonyl coA binding pocket has occurred in one of the alleles. The docking experiment was performed using the protein model for both the alleles as the template and the malonyl coA as the ligand. The docking score for both the alleles was variable. The CHS putative wild-type allele which contained glutamic acid (Fig 9A) was found to have a docking score of -11.87. The mutant allele having the amino acid alanine (Ala) as the substitution in place of glutamic acid (Glu) in the malonyl coA binding pocket (Fig 9B) was found to have less affinity towards the ligand malonyl coA having the docking score of -10.35.

### Phenotyping of Powdery Mildew infected F_1_ mulberry population

**Fig 10:**
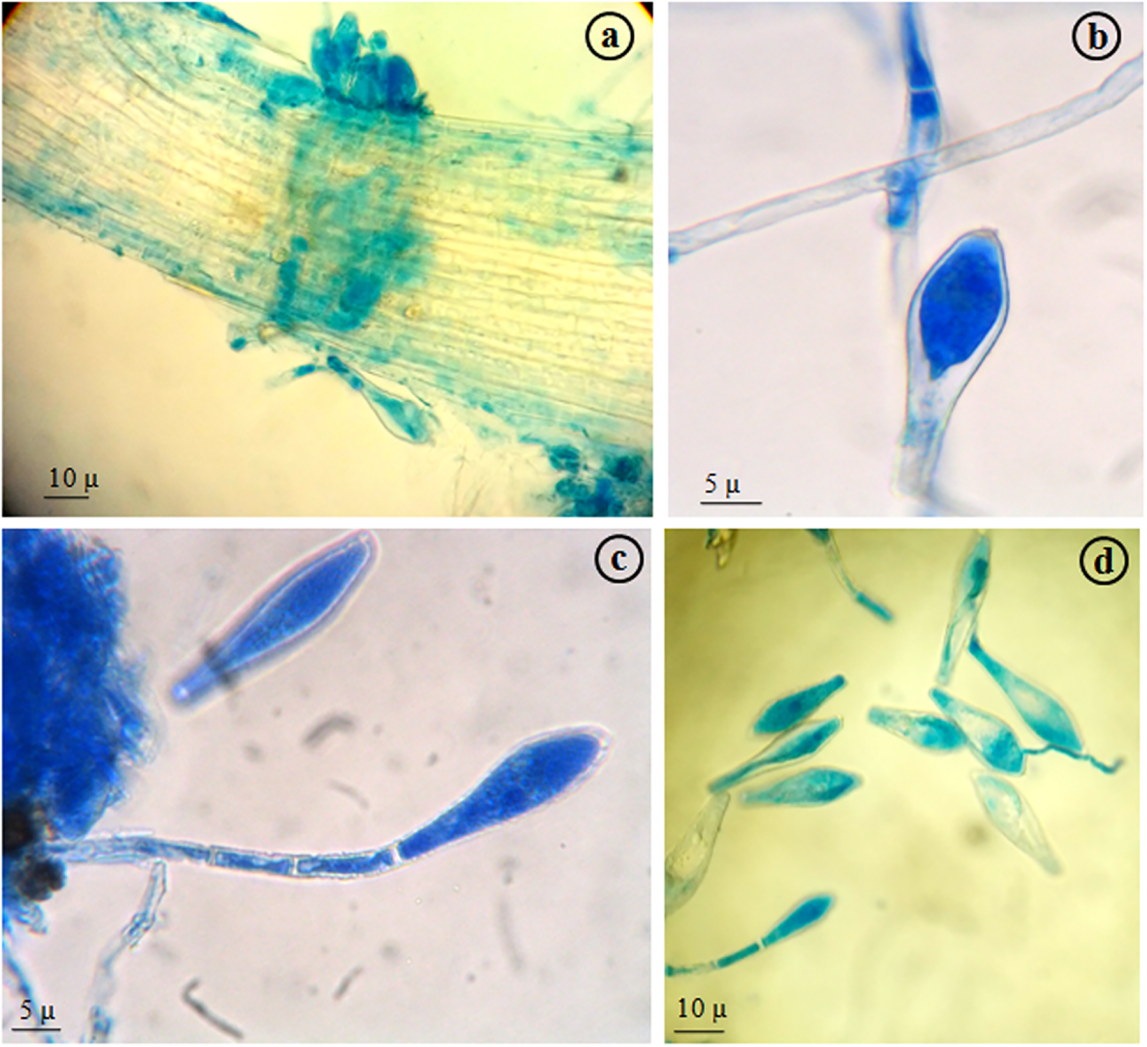
Microscopic images of *Phyllactinia* spp., the causal organism of Powdery Mildew disease in mulberry. a: fungal progression in infected leaf, b: a single conidium, c: conidium with characteristic conidiophores, d: multiple conidia.

The causal organism was identified from the infected leaves through microscopic observations (Fig 10A–10D). Conidia were 47.2–66.7 × 14.2–22.1 μm, single-celled, hyaline and club-shaped occurring singly on unbranched, straight and cylindrical conidiophores. Only asexual stage was observed as the pathogen did not reach the end of the growing season.Based on the characteristics of asexual state and host specification, the fungus was identified as *Phyllactinia* sp.

**Fig 11:**
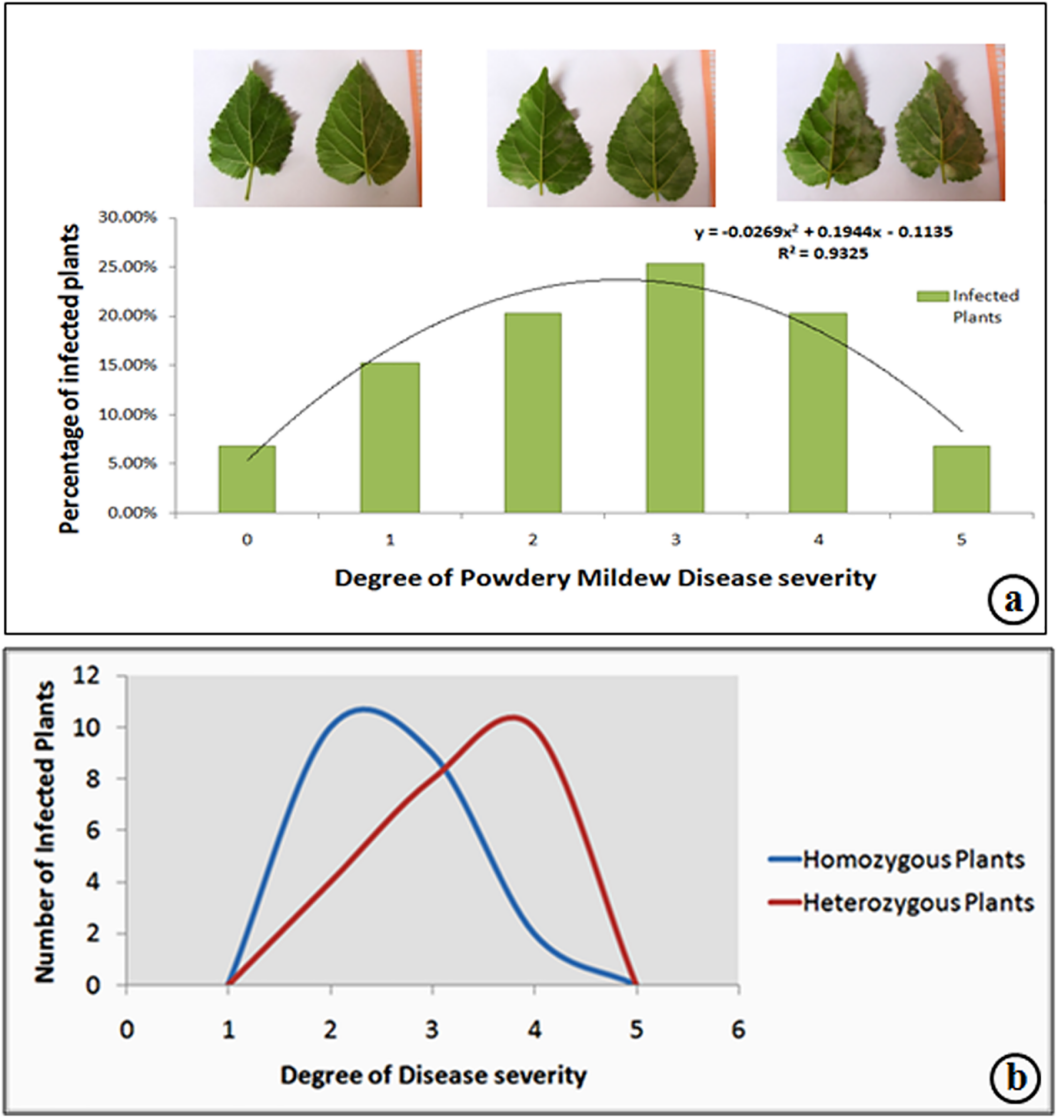
a: Distribution curve of Powdery Mildew disease severity in the F_1_ population infected with *Phyllactinia* spp. b: Skewing of mean disease score in homozygous and heterozygous population with respect to the *CHS* locus.

The degree of disease severity varied in the heterozygous and homozygous plants with respect to *CHS* locus (Fig 11A). The maximum number of homozygous plants restricted the severity of the infection (mean disease score 2.5), while the disease severity was high in most of the heterozygous plants (mean disease score 4.0). The statistical distribution curve showed a distinct skewing of mean of disease severity between homozygous and heterozygous plants with respect to *CHS* locus (Fig 11B).

## Discussion

Prospect of chalcone synthase as a useful candidate gene is explored in recent times in few plant systems [26–28]. Mulberry being a non-model plant system has significantly less validated genetic information in the genomic databases. A very recent report of characterization and functional analysis of 4-Coumarate-CoA Ligase genes in mulberry [29] indicates that the genes of this pathway can be used as potent candidates for the development of molecular markers. In the present work, we have demonstrated the presence of two allelic form of *CHS* gene in an F_1_ recombinant population of a mulberry hybridization program. The study resulted in the development of an SNP-based CAPS marker to discriminate and identify both the alleles of *CHS* gene.

In the present work, we have successfully isolated, cloned and analysed the partial genomic and coding sequence as well as the full-length sequence of both the alleles of *CHS* gene. The coding sequence of both the alleles revealed several distinct SNPs in the three genotypes (Fig 4). The presence of an internal restriction endonuclease site of EcoR1 in the partial genomic sequence of one of the allele of *CHS* gene helped us to distinguish between the alleles using the developed SNP-based CAPS marker. In the study materials of the present work comprising of both male and female parent and fifty-five random F_1_ recombinants, the heterozygous individual showed both the *CHS* alleles—one allele (*chs*) containing the internal EcoRI site, while the other allele lacks the internal restriction site. Hence, the CAPS profile of the heterozygous individuals showed the presence of three restriction digested fragments- two fragments at 500bp resulting from *chs* allele, and one undigested fragment at ~1kb. The homozygous individuals, on the other hand, due to the presence of identical alleles (both without restriction sites) resulted in a prominent undigested 1Kb product (Fig 5). The credibility of this SNP-based CAPS marker has also been demonstrated in the F_1_ population, as the recombinants show a 1:1 segregation ratio between homozygous and heterozygous locus of *CHS* gene. The male parent (V1) is heterozygous for the *CHS* locus while the female parent (Kajli-OP) is homozygous. Of the ten mulberry varieties the CHS locus was in homozygous state in six genotypes (C776, S30, C763, S1635, S1, and C2028). This finding can be extrapolated with the long successful cultivation history of these genotypes as varieties in different parts of mulberry growing areas in India.

The segregation pattern of CAPS marker is further found to be co-relatable with the results of chalcone synthase enzyme assay, as the mean enzyme activity differed significantly between homozygous and heterozygous individuals in the population. Hence, it can be assumed that the lower chalcone synthase activity in the heterozygous individuals is due to the presence of the identified chs allele that makes it as an unwanted one. To validate these findings, we performed homology modelling and docking studies for both the *CHS* alleles. The objective was to determine whether the SNP in the coding sequence resulting in amino acid changes have any effect on the three-dimensional structure of the CHS protein or whether they affect the substrate binding affinity of the CHS enzyme, which can lead to decreased enzyme activity. The results obtained from the homology modelling analysis showed that the substitutions in the amino acid residues of *chs* allele (Fig 3) do not affect the overall folding and three dimensional structure of the functional protein. Apparently no such structural changes or distortions were found in the functional protein from both the alleles. However, the docking study results showed significant difference in the free energy change for the binding of both the functional CHS protein to malonyl coA which is one of its substrate. The chs protein showed less affinity for malonyl coA, this can be attributed to the fact that in the chs protein the Glu 99 and Glu 118 has been substituted with Ala in the malonyl coA binding site. This change from a negatively charged hydrophilic amino acid Glu to a hydrophobic uncharged amino acid Ala lead to the decreased affinity for the binding of malonyl-CoA.

The findings of the present study demonstrated the importance of the developed CAPS marker as it defines the *chs* allele and will be helpful to eliminate the individuals having this allele in the preliminary screening of any breeding programme of mulberry targeting towards the development of stress tolerant genotypes. The phenotyping of the F_1_ population provided the circumstantial evidence for the probable association between the marker and the degree of Powdery Mildew disease progression in mulberry. Mulberry being an extremely out breeding and heterozygous tree crop lacks the presence of pure line plants which poses a major problem in selection of parents for mulberry breeding. This study further depicts the consequence of hybridization between parents of unknown genetic information. The genotype (Gen1) resulting from the present hybridization and selection is a proven high leaf yielding one, awaiting release as a variety. However, the presence of the unwanted *chs* allele, which it has inherited from its male parent (V1), may result in its susceptibility to diverse abiotic and biotic stress in future. Furthermore, the usefulness of wild and landraces as the repertoire of desirable alleles is substantiated by the presence of favourable *CHS* allele in the homozygous state in an open pollinated landrace (Kajli-OP) used as the female parent in the current breeding programme.

## References

[1] Arora V, Ghosh MK, Bindroo BB, Gangopadhyay G. Phenomic analyses of indigenous and exotic accessions of Mulberry (*Morus* spp.). Int. Res. J. Biological Sci. 2014; 3:40–48.

[2] Rao AA. Conservation status of mulberry genetic resources in India. Paper contributed to Expert Consultation on Promotion of Global Exchange of Sericultural Germplasm Resources, Satellite session of 19th ISC Congress. Bangkok, Thailand. 2002; 21–25.

[3] Das M, Chauhan H, Chhibbar A, Haq QMR, Khurana P. High-efficiency transformation and selective tolerance against biotic and abiotic stress in mulberry, *Morus indica* cv. K2, by constitutive and inducible expression of tobacco osmotin. Transgenic Res. 2011; 20: 231–246. doi: 10.1007/s11248-010-9405-6 20549349 10.1007/s11248-010-9405-6

[4] Vijayan K. Molecular Markers and Their Application in Mulberry Breeding. Int.J.Indust. Entomol. 2007; 15: 1–11.

[5] Bargsten JW, Nap J-P, Sanchez-Perez GF, van Dijk ADJ. Prioritization of candidate genes in QTL regions based on associations between traits and biological processes. BMC Plant Biology. 2014; 14:330 doi: 10.1186/s12870-014-0330-3 mailto:aaltjan.vandijk@wur.nl 25492368 10.1186/s12870-014-0330-3 PMC4274756

[6] Schröder J. A family of plant-specific polyketide synthases: Facts and predictions. Trends in Plant Science. 1997; 2: 373–378.

[7] Zhao S, Park CH, Li X, Kim YB, Yang J, Sung B. et al Phenylpropanoid and Triterpenoid Biosynthetic Genes in Mulberry (*Morus alba* L.). J. Agric. Food Chem. 2015; 63: 8622–8630. doi: 10.1021/acs.jafc.5b03221 26343778 10.1021/acs.jafc.5b03221

[8] Winkel-Shirley B. Biosynthesis of flavonoids and effects of stress. Current Opinion in Plant Biology. 2002; 5: 218–223. 11960739 10.1016/s1369-5266(02)00256-x

[9] Taylor L P, Grotewold E. Flavonoids as developmental regulators. Current Opinion in Plant Biology. 2005; 8: 317–323. doi: 10.1016/j.pbi.2005.03.005 15860429 10.1016/j.pbi.2005.03.005

[10] Lillo C, Lea U S, Ruoff P. Nutrient depletion as a key factor for manipulating gene expression and product formation in different branches of the flavonoid pathway. Plant Cell & Environment. 2008; 31: 587–601. 10.1111/j.1365-3040.2007.01748.x 18031469

[11] Päsold S, Siegel I, Seidel C, Ludwig-Müller J. Flavonoid accumulation in *Arabidopsis thaliana* root galls caused by the obligate biotrophic pathogen *Plasmodiophora brassicae*. Molecular Plant Pathology. 2010; 11: 545–562. doi: 10.1111/j.1364-3703.2010.00628.x 20618711 10.1111/j.1364-3703.2010.00628.x PMC6640481

[12] Dao TTH, Linthorst HJM, Verpoorte R. Chalcone synthase and its functions in plant resistance. Phytochem Rev. 2011; 10: 397–412. doi: 10.1007/s11101-011-9211-7 21909286 10.1007/s11101-011-9211-7 PMC3148432

[13] Fini A, Brunetti C, Di Ferdinando M, Ferrini F, Tattini M. Stress-induced flavonoid biosynthesis and the antioxidant machinery of plants. Plant Signalling & Behaviour. 2011; 6: 709–711. 10.4161/psb.6.5.15069 PMC3172844 21448007

[14] Martens S, MithöferA. Flavones and flavone synthases. Phytochemistry. 2005; 66: 2399–2407. doi: 10.1016/j.phytochem.2005.07.013 16137727 10.1016/j.phytochem.2005.07.013

[15] Qi X, Shuai Q, Chen H, Fan L, Zeng Q, He N. Cloning and expression analyses of the anthocyanin biosynthetic genes in mulberry plants. Mol Genet Genomics. 2014; 289: 783–793. doi: 10.1007/s00438-014-0851-3 24748075 10.1007/s00438-014-0851-3

[16] Arora V, Ghosh MK, Gangopadhyay G. SSR Markers for assessing the hybrid nature of two high- yielding mulberry varieties. Int. J. Genetic Engineering Biotechnology. 2014; 5:191–196.

[17] Obinata N, Yamakawa T, Takamiya M, Tanaka N, Ishimaru K, Kodama T. Effects of salicylic acid on production of procyanidin and anthocyanin in cultured grape cells. Plant Biotechnology. 2003; 20:105–111.

[18] Moustafa E, Wong E. Purification and properties of chalcone-flavanone isomerase from soyabean seed. Phytochemistry. 1967; 6: 625–632.

[19] Guex N, Peitsch MC. SWISS-MODEL and the Swiss- Pdb Viewer: an environment for comparative protein modelling. Electrophoresis. 1997; 18: 2714–2723. doi: 10.1002/elps.1150181505 9504803 10.1002/elps.1150181505

[20] Liang J, Edelsbrunner H, Woodward C. Anatomy of protein pockets and cavities: measurement of binding site geometry and implications for ligand design.Protein Sci. 1998; 7: 1884–1897. doi: 10.1002/pro.5560070905 9761470 10.1002/pro.5560070905 PMC2144175

[21] Bhan N, Li L, Cai C, Xu P, Linhardt RJ, Koffas Mattheos AG. Enzymatic formation of a resorcylic acid by creating a structure-guided single-point mutation in stilbene synthase. Protein Sci. 2015; 24: 167–173. doi: 10.1002/pro.2600 25402946 10.1002/pro.2600 PMC4315654

[22] Marchler-Bauer A, Derbyshire MK, Gonzales NR, Lu S, Chitsaz F, Geer LY et al CDD: NCBI's conserved domain database. Nucleic Acids Res. 2015; 43:222–226. 10.1093/nar/gku1221 PMC4383992 25414356

[23] Marchler-Bauer A, Lu S, Anderson JB, Chitsaz F, Derbyshire MK, DeWeese-Scott C et al CDD: a Conserved Domain Database for the functional annotation of proteins. Nucleic Acids Res. 2011; 39: 225–229. 21109532 10.1093/nar/gkq1189 PMC3013737

[24] Marchler-Bauer A, Anderson JB, Chitsaz F, Derbyshire MK, DeWeese-Scott C, Fong JH. et al CDD: specific functional annotation with the Conserved Domain Database. Nucleic Acids Res. 2009; 37: 205–210. 10.1093/nar/gkn845 PMC2686570 18984618

[25] Marchler-Bauer A, Bryant SH. CD-Search: protein domain annotations on the fly. Nucleic Acids Res. 2004; 32: 327–331. 10.1093/nar/gkh454 PMC441592 15215404

[26] Cardoso DC, Martinati JC, Giachetto PF, Vidal RO, Carazzolle MF, Padilha L. et al Large-scale analysis of differential gene expression in coffee genotypes resistant and susceptible to leaf miner–toward the identification of candidate genes for marker assisted-selection BMC Genomics. 2014; 15: 1–20. 24460833 10.1186/1471-2164-15-66 PMC3924705

[27] Deng N, Chang E, Li M, Ji J, Yao X, Bartish IV. et al Transcriptome Characterization of *Gnetum parvifolium* Reveals Candidate Genes Involved in Important Secondary Metabolic Pathways of Flavonoids and Stilbenoids. Frontiers in Plant Science. 2016; 7: 1–15. 26973657 10.3389/fpls.2016.00174 PMC4778121

[28] Xin M, Wang X, Peng H, Yao Y, Xie C, Han Y. et al Transcriptome Comparison of Susceptible and Resistant Wheat in Response to Powdery Mildew Infection. Genomics Proteomics Bioinformatics. 2012; 10: 94–106. doi: 10.1016/j.gpb.2012.05.002 22768983 10.1016/j.gpb.2012.05.002 PMC5054165

[29] Wang CH, Yu J, Cai YX, Zhu PP, Liu CY, Zhao AC. et al Characterization and Functional Analysis of 4-Coumarate: CoA Ligase Genes in Mulberry. PLOS ONE. 2016; doi: 10.1371/journal.pone.0155814 1–20 10.1371/journal.pone.0157414 PMC4898743 27276057

